# The Humoral and Cellular Immune Response to the Administration of OrthopoxVac Vaccine to Volunteers

**DOI:** 10.32607/actanaturae.27654

**Published:** 2025

**Authors:** S. N. Shchelkunov, E. Yu. Prudnikova, A. A. Shestakova, S. N. Yakubitskiy, S. A. Pyankov, A. E. Nesterov, S. V. Usova, M. P. Bogryantseva, E. A. Nechaeva, T. V. Tregubchak, A. P. Agafonov

**Affiliations:** State Research Center of Virology and Biotechnology VECTOR, Rospotrebnadzor, Koltsovo, Novosibirsk region, 630559 Russia

**Keywords:** smallpox, monkeypox, vaccinia virus, vaccination, antibodies, T-cells

## Abstract

OrthopoxVac, a fourth-generation smallpox vaccine, was the first of its kind registered worldwide in 2022, and it has been shown to be both safe and to induce only a mild reaction. A six-month clinical study confirmed its immunogenicity as compared to the first-generation live smallpox vaccine. Our study aimed to determine the levels of specific humoral and T-cell immune responses in volunteers following intradermal OrthopoxVac vaccine administration either in a single dose of 107 PoFU or in two doses of 106 PoFU, at 1.5, 3, and 5 years after initial vaccination. Following the immunization of volunteers with the OrthopoxVac vaccine at a dosage of 107 PoFU, the T-helper response remained at a relatively high level for three years, before it significantly dropped. Administration of the same vaccine twice at a dose of 106 PoFU resulted in a considerable decrease in the level of T-helpers, after 1.5 years. Additionally, some patients exhibited a reduction in viral neutralizing antibody (VNA) titers after 1.5 years of OrthopoxVac vaccine administration. When OrthopoxVac was administered at a dosage of 107 PoFU, no substantial differences were noted between groups at the 1.5-, 3-, and 5-year marks. In contrast, in the groups receiving two doses of 106 PoFU, VNA titers showed a significant reduction after 1.5 years. These findings indicate that a single intradermal dose of 107 PoFU of the OrthopoxVac vaccine elicits a significant and lasting immune response involving both antibodies and T-cells for a minimum of three years.

## INTRODUCTION

Smallpox, a highly toxic, deadly, and extremely contagious human infectious disease, is also the only disease eradicated amongst humans through a global vaccination and epidemic surveillance campaign by the World Health Organization (WHO). This achievement remains one of the greatest triumphs of medical science [1].

The smallpox eradication program extensively utilized first-generation vaccines, which were mainly derived from the vaccinia virus (VACV). The virus was propagated on the skin of live animals, predominantly calves, with sheep, buffalo, and rabbits being used to a lesser extent. A major disadvantage of these vaccines has remained the high rate of serious postvaccination complications, especially in people with immunodeficiencies, atopic dermatitis, and elderly individuals who have never received the smallpox vaccine [1, 2].

Adverse reactions, with varying degrees of prevalence and intensity, occur in approximately 20–30% of individuals that are vaccinated with the first-generation smallpox vaccine. The most frequently reported adverse reactions are low-grade fever, headache, lymph node swelling, skin inflammation, and fatigue. Significantly fewer individuals who receive the vaccine experience severer conditions, such as eczema, generalized or progressive vaccinia, encephalitis, or myopericarditis. Serious adverse events are observed in only a small fraction of vaccinated individuals, up to several hundreds per million, and fatalities amount to one or two patients per million [1, 3]. Given the severe post-vaccine complications that had accompanied the classical live vaccine and after confirmation of the eradication of smallpox in 1980, the WHO issued a strong recommendation to all nations that they discontinue vaccination against the infection [1, 2].

It is important to note that natural reservoirs harbor zoonotic orthopoxviruses closely related to the variola virus, including the monkeypox virus, cowpox virus, and other viruses that can infect humans [4]. Due to the cessation of smallpox vaccination, a considerable number of people, mainly those under 45 years of age, are no longer protected against orthopoxvirus infections. In the past few years, multiple outbreaks of zoonotic orthopoxvirus infections have been reported in human populations across geographical regions [2, 4]. Of significant concern has been the incidence of human monkeypox virus infections that resulted in an epidemic of this orthopoxvirus disease that spread across all continents between 2022 and 2023, affecting populations in over 100 countries [5]. At the moment, the primary area of concern regarding human monkeypox transmission is Africa [6]. Thus, renewed and intensified attention should now be focused on the possibility of a resurgence of smallpox or a comparable, dangerous disease that is a result of the natural evolution of zoonotic orthopoxvirus infectious agents [7, 8].

To lower the risk of widespread epidemics that stem from localized outbreaks and the natural evolution of a highly pathogenic human orthopoxvirus, researchers should prioritize the development of safe, new-generation live vaccines based on VACV. These factors highlight the scientific and practical significance of, as well as the urgency for; an updated strategy in the realm of vaccine prophylaxis against infections caused by orthopoxviruses.

Advancements in genetic engineering techniques have enabled the design of modified VACV variants through the targeted insertion of sequences into the viral genome, or by deleting or disrupting specific virulence genes [9, 10], while maintaining the genes essential for viral replication in cell culture. Deactivating virulence genes can markedly diminish the pathogenic attributes of VACV. A particularly promising avenue of research involves the development of highly attenuated variants of VACV through genetic engineering which exhibit an immunogenicity and protective efficacy similar to that of the original smallpox vaccine, but with significantly reduced pathogenicity.

OrthopoxVac, our fourth-generation live vaccine, is a variant designed to protect against smallpox and other orthopoxvirus infections. This vaccine uses the VACΔ6 strain, which harbors six gene disruptions (*C3L*, *N1L*, *J2R*, *A35R*, *A56R, *and *B8R*) and is cultivated in the 4647-cell culture [2, 11].

It is important to study the length of the immune response after vaccination in people who have received the OrthopoxVac vaccine. The results should be compared to the immune response triggered by the live smallpox vaccine (LSV) earlier used in Russia [12]. Such a study will provide insights into the necessity for and timing of revaccination using the new fourth-generation vaccine.

This research aimed to delve into post-registration data from the OrthopoxVac vaccine (a live culture vaccine for the prevention of smallpox and related orthopoxvirus infections based on the vaccinia virus), focusing on the level and duration of the immune protection provided by both antibodies and T cells.

## EXPERIMENTAL PART

**Overall study design**

**Fig. 1:**
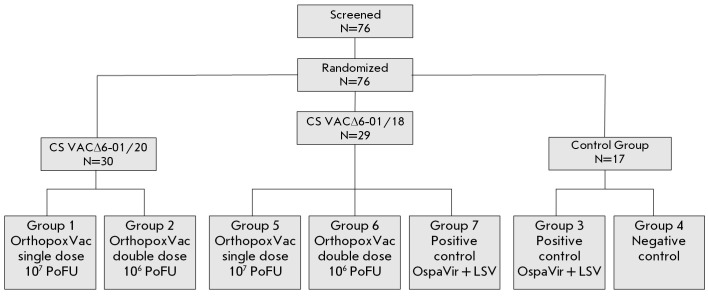
The scheme to distribute volunteers by groups

A randomized, comparative, parallel-group study was performed, enrolling 76 subjects (male and female) aged between 25 and 40 years, who satisfied the inclusion criteria, met no exclusion criteria, and had prior participated in Phase I (CS VACΔ6-01/18) and Phase II/III (CS VACΔ6-01/20) clinical studies (CS) of OrthopoxVac vaccine (*Fig. 1*).

Group 1 comprised 15 healthy volunteers (7 men and 8 women) enrolled in the VACΔ6-01/20 clinical study who received a single intradermal vaccination with the OrthopoxVac vaccine at a dose of 10^7^ PoFU;

Group 2 comprised 15 healthy volunteers (6 men and 9 women) enrolled in the VACΔ6-01/20 clinical study and vaccinated twice intradermally with the OrthopoxVac vaccine at a dose of 10^6^ PoFU at intervals of 28 days;

Group 3 (positive control, PC) comprised 7 healthy volunteers (4 men and 3 women) who had worked with viruses of the genus Orthopoxvirus and were vaccinated using a two-stage method with the inactivated smallpox vaccine OspaVir and, after 7 days, again with a live smallpox vaccine based on the strain L-IVP VACV (“Microgen”, Russia) as described in the previous paper [13] (OspaVir + LSV, 2020);

Group 4 (negative control, NC) consisted of 10 healthy volunteers (6 men and 4 women) who had never been previously immunized with any smallpox vaccine, had had no contact with patients immunized with a smallpox vaccine, and had never handled viruses belonging to the genus Orthopoxvirus;

Group 5 comprised 9 healthy volunteers (3 men and 6 women) who had participated in the VACΔ6- 01/18 clinical study and were administered a single intradermal vaccination of the OrthopoxVac vaccine, at a dosage of 10^7^ PoFU;

Group 6 comprised 10 healthy volunteers (7 men and 3 women) enrolled in the clinical study VACΔ6-01/18 who had received two intradermal vaccinations of the OrthopoxVac vaccine (10^6^ PoFU) at 28-day intervals; and

Group 7 (PC) comprised 10 healthy volunteers (5 men and 5 women) who had participated in the VACΔ6-01/18 clinical study and were vaccinated using the two-stage method of OspaVir + LSV.

Each patient provided written informed consent before inclusion in the study.

**Viruses, cell culture**

The research employed the L-IVP [9] and VACΔ6 VACV [11] strains and the CV-1 African green marmoset kidney cell line, which were sourced from the cell culture collection of the State Research Center of Virology and Biotechnology “ector” Rospotrebnadzor.

**Collection of blood samples from the volunteers**

Blood sampling was performed from the ulnar vein in the hospital and inoculation room with observance of aseptic and antiseptic rules. A volume of 30–35 mL of blood was drawn during a single collection, using vacuum tubes. This work was performed at the clinical base of the Federal State Budgetary Healthcare Institution, Medical and Sanitary Unit No. 163 of the Federal Medical and Biological Agency of Russia.

The study was approved by the Ethical Committee of the State Research Center of Virology and Biotechnology “Vector”, Rospotrebnadzor (Protocol No. 10 of the Ethical Committee meeting, February 14, 2024).

For the assessment of humoral immunity, serum was obtained from blood samples by precipitating the formed elements via centrifugation for 10 minutes at 1,000 × g and 4°C. The resulting serum samples were heat-inactivated at 56°C for 30 minutes and stored at -20°C.

**Immunoenzymatic analysis of blood sera**

The titers of specific antibodies were determined by ELISA using the “Vector ELISA Pox-IgG reagent kit for the immunoenzymatic detection of class G antibodies to poxvirus antigens” (Registration Certificate No. RZN 2022/15638), in accordance with the manufacturer’ instructions [14].

**Determination of the viralneutralizing antibody titer in sera**

A plaque reduction assay of the VACV strain L-IVP in the CV-1 cell culture was used to determine the titer of virus-neutralizing antibodies (VNA). Four serial two-fold dilutions of a volunteer serum samples were prepared for the assay starting from 1 : 10 up to 1 : 80. Additional double dilutions, ranging from 1 : 160 to 1 : 1,280, were utilized to specify VNA titers for samples that demonstrated serum neutralizing activity beyond 1 : 80. Subsequently, an equal volume of the VACV dilution, with a titer of approximately 400 PFU/mL (approximately 40 PFU/well), was added to the prepared serum dilutions. The resulting mixtures were incubated at 37°C for 1 h. All the serum and virus dilutions were prepared using a maintenance medium: a DMEM/F-12 nutrient medium (1 : 1) supplemented with a 2% fetal bovine serum (FBS), 100 IU/mL penicillin, and 100 μg/mL streptomycin.

Subsequently, 200 µL of each serum-virus mixture was applied onto a 90–00% confluent monolayer of CV-1 cells grown in a 24-well culture plate, using three wells per serum dilution. Viral adsorption was carried out for 1 h at 37°C in a humidified atmosphere containing 5% CO_2_. After the adsorption period, the maintenance medium (1 mL/well) was added and the cells were incubated for an additional 48 h at 37°C and 5% CO_2_. Following the incubation period, the culture medium was removed and the cells were fixed and stained for 15 minutes by applying a solution of 0.2% crystal violet in a 9.6% ethanol aqueous solution containing 2% formaldehyde (approximately 0.2 mL/well). Subsequently, the dye was removed and the culture plate was dried at room temperature.

The number of plaques, representing the foci of cellular monolayer destruction with distinctive white spots on a blue background, was quantified in the CV-1 cell culture monolayer, and the serum dilutions that inhibited 50% PFU formation compared to the number of PFU in the negative control group (nonimmune serum wells) were determined. Calculations were performed using the Spearman-Kärber method, and the results were expressed as the 50% plaquereduction neutralization titer.

**Peripheral blood mononuclear cell (PBMC) isolation**

Venous blood was obtained from volunteers and collected in heparinized tubes (10 U/mL). PBMCs were isolated in a ficoll density gradient of 1.077 g/mL. The collected cell suspension was washed three times with the DMEM/F12 medium supplemented with 5% FBS, and the cells were pelleted via centrifugation at 350 g for 15 minutes at a temperature of (10 ± 2)°C. The cellular sediment was resuspended in the DMEM/F12 medium supplemented with 15% FBS. Following this, a cell suspension at a concentration of 10 million cells/mL was prepared and 100 μL of the suspension was added to the wells of a 96-well flat-bottom culture plate (1 × 10^6^ cells/well).

**Intracellular staining of cells for cytokines**

The cell-mediated immune response was evaluated via intracellular cytokine staining following stimulation of PBMC with antigen. Each sample was evaluated using the following conditions: unstimulated cells (background control), cells stimulated with virus- containing material (purified vaccinia virus strain VACΔ6, 4.0 µg of total protein), and a positive control comprising cells stimulated with 50 ng/mL phorbol 12-myristate 13-acetate (Sigma-Aldrich, USA) and 0.5 µg/mL ionophore (Calcium Ionophore A23187; Sigma-Aldrich, USA). The cells were incubated at 37°C in a 5.0% CO_2_ atmosphere for 8 h, followed by the addition of GolgiPlug (BD Biosciences, USA) to each well in accordance with the manufacturer’s instructions, and followed again by additional overnight incubation at 37°C within a 5.0% CO_2_ atmosphere. After stimulation, the cells underwent washing using a phosphate-buffered saline solution with a 2% casein hydrolysate. Next, the cells were stained for 40 min at 4°C with the Fixable Viability Stain 780 dye and the monoclonal antibodies CD3 (clone SK7, BV786), CD4 (clone RPA-T4, PerCP-Cy 5.5), CD8 (clone RPA-T8, Alexa Fluor 700), and CD45RA (clone HI100, BV510), CCR7 (CD197) (clone 3D12, PE-Cy7) (BD Biosciences). Subsequently, the cells were washed three times using a 2% phosphate-salt buffer solution and incubated for 20 minutes with 100 μl of the Fixation/ Permeabilization solution (BD Biosciences). Following incubation, the samples were washed thrice using 1× wash buffer (BD Perm/Wash™Buffer, BD Biosciences) and stained for 40 minutes with monoclonal antibodies specific to interleukin-2 (IL-2, clone MQ1 17H12, APC), tumor necrosis factor (TNF, clone MAb11, PE), and interferon-γ (IFN-γ, clone B27, BV421, BD Biosciences). After washing three times with 1× wash buffer, the cells were fixed in 300 μL of 1× buffer (BD CellFix, BD Biosciences). The fixed cells were assessed using an ACEA NOVOCite Quanteon 4025 flow cytometer (Agilent Technologies, USA). Data analysis was performed with the NovoExpress software version 1.5.0.

**Fig. 2:**
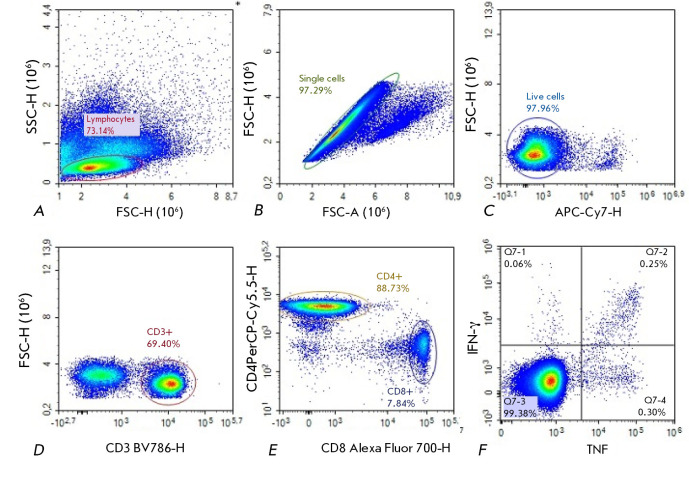
Gating strategies for the identification of the principal T cell subsets (refer to text for details)

The cytometric analysis used the following gating strategy (*Fig. 2*). The lymphocyte population was first identified based on forward and side scatter characteristics (*Fig. 2A*). Subsequently, singletons (single cells) were isolated: the abscissa represents the integral signal of direct light scattering, and the ordinate represents the peak signal of direct light scattering (*Fig. 2B*). Next, live cells negative for APC-Cy7 were isolated from single cells (*Fig. 2C*). BV786-positive T cells were gated according to the CD3 expression level (*Fig. 2D*). Cytotoxic T lymphocytes (CD3^+^CD8^+^ phenotype) were differentiated from helper T lymphocytes (CD3^+^CD4^+^ phenotype) using the histogram presented in *Fig. 2E*. The graph in *Fig. 2F* depicts cytokine-positive T helper cells, specifically those producing the tumor necrosis factor (TNF) and interferon- gamma (IFN-γ). The graph in *Fig. 2F* illustrates T-helper cells, identified by their positivity for the TNF and IFN-γ cytokines.

**Statistical data analysis**

Statistical analysis was performed using one-factor analysis of variance (ANOVA) for three or more groups. A comparison of the two groups was conducted using the F-criterion. Statistical significance was established for result variations at *p * < 0.05.

## RESULTS

**Detection of VACV-specific antibodies by ELISA**

A reliable condition of vaccination efficacy is to use the antibody titers in the control group samples as a comparative benchmark: the negative control (NC) group, comprising samples from volunteers who had until then never been vaccinated with smallpox vaccines, had no contact with vaccinated patients, and had had no occupational exposure to orthopoxviruses and the positive control (PC) group, which consists of samples from volunteers vaccinated with a first-generation vaccine, collected 3 and 5 years post-vaccination.

In the experimental groups of phase II/III clinical studies, three years post-vaccination, the percentage of volunteers exhibiting ELISA titers ≥ 1 : 100 was 86.7% following a single 10^7^ PoFU dose of OrthopoxVac, and 92.8% after two administrations of a 10^6^ PoFU dose. After five years, no serum samples from the phase I clinical study volunteer groups exhibited titers below 1 : 100.

The geometric mean titer (GMT) of specific IgG detected by ELISA was established to be 46 in the NC group, with an error range of 36 to 58 for the 95% confidence interval.

**Fig. 3:**
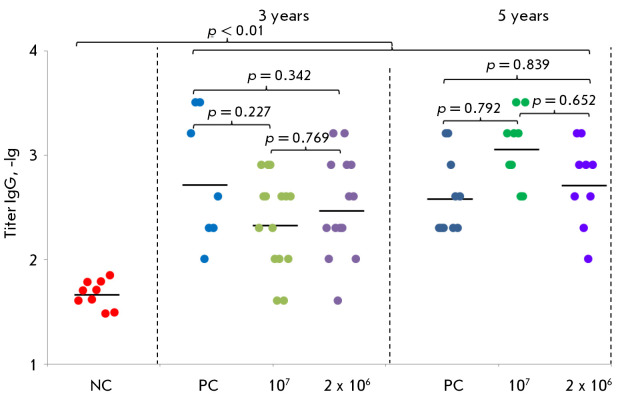
Logarithms of the ELISA titers of specific IgG to VACV antigens in the blood sera of volunteers from clinical studies of the OrthopoxVac vaccine. NC group (comparison group, negative control) – volunteers who had not been vaccinated with smallpox vaccines, had not been in contact with patients vaccinated with smallpox vaccines, and did not work with viruses of the genus Orthopoxvirus; PC group (positive control) – volunteers vaccinated by the twostage method with the smallpox inactivated OspaVir vaccine and after 7 days with a live smallpox vaccine based on the L-IVP strain (Microgen); 10^7^ group – volunteers vaccinated once intradermally with the OrthopoxVac vaccine at a dose of 10^7^ PoFU/0.2 mL; 2 × 10^6^ group – volunteers vaccinated twice at 28-day intervals, intradermally at a dose of 10^6^ PoFU/0.2 mL. The significance of the differences between the groups was determined by the F criterion. Each point represents a single volunteer. Horizontal lines denote GMT values for each group

The remaining control and experimental groups exhibited significantly different values, with considerably expanded error margins. For example, three years post-vaccination, the GMT values were 212 (121–372), 292 (155–555), and 518 (137–1952) in the 10^7^ PoFU, 2 × 10^6^ PoFU, and PC groups, respectively. Five years post-vaccination, the GMT values in the same groups were 1131 (619–2065), 510 (251–1038), and 379 (204–704), respectively. A logarithmic interpretation of the obtained data is presented in *Fig. 3*.

Statistically significant differences were observed only within the NC group, relative to the other three groups at both the three-year and five-year post-vaccination intervals (*Fig. 3*). In the remaining pairs of groups, the differences are not significant.

**Determination of virus-neutralizing antibody titers in the VACV neutralization assay**

The conferring of protective immunity against smallpox and other orthopoxvirus infections is significantly influenced by virus-neutralizing antibodies [15, 16]. The measured VNA titer can depend on the specific virus-cell culture pair and the details of the methodology used. Therefore, a surefire criterion for evaluating vaccination efficacy for this indicator is to use the first-generation vaccine as a control. Its efficacy against smallpox has been previously demonstrated.

**Fig. 4:**
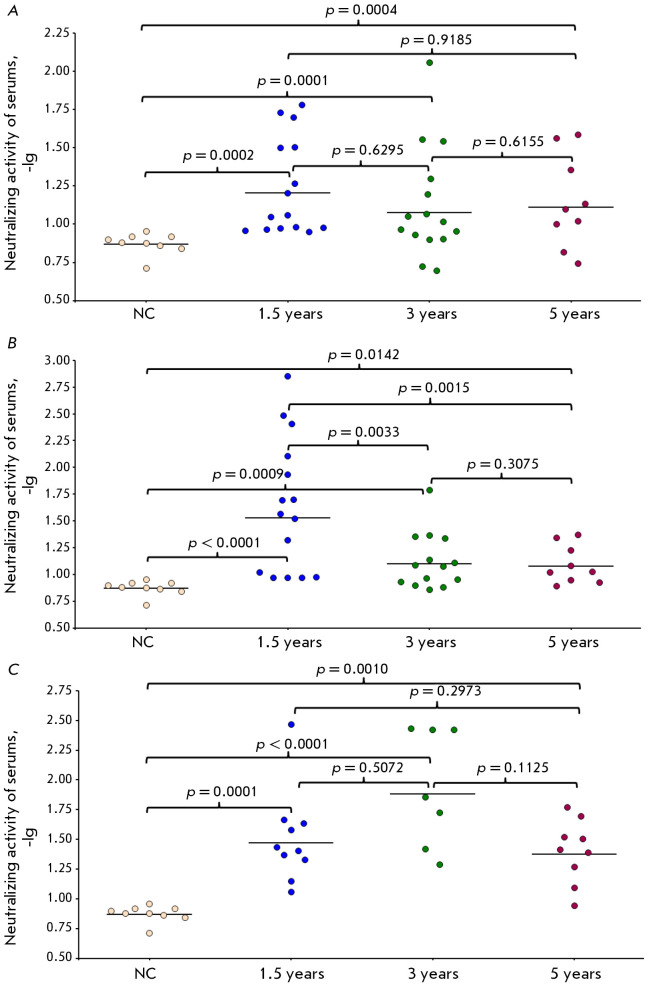
The neutralizing activity of the blood sera of volunteers vaccinated in phases I and II/III clinical studies of the Orthopox- Vac vaccine. Virus-neutralizing antibody titers were assessed via the plaque reduction assay of VACV (strain L-IVP) on CV-1 cell cultures. The data are presented in the form of -lg, with each point representing a single volunteer and horizontal lines indicating the levels of GMT of antibodies in the groups. The significance of the differences between the groups was determined by the F criterion. Presented are data on VNA titers at 1.5, 3, and 5 years post-vaccination for: (*A*) group – volunteers vaccinated once intradermally with the Orthopox- Vac vaccine at a dose of 10^7^ PoFU/0.2 mL; (*B*) group – volunteers vaccinated twice with an interval of 28 days intradermally with the OrthopoxVac vaccine at a dose of 10^6^ PoFU/0.2 mL; (*C*) group (positive control) – volunteers vaccinated with a two-stage technique: inactivated smallpox vaccine and then Smallpox live vaccine; NC group (comparison group, negative control) – volunteers who were not vaccinated with smallpox vaccines, were not in contact with patients vaccinated with smallpox vaccines, and did not work with viruses of the genus Orthopoxvirus

Our findings (*Fig. 4*) demonstrate that 1.5 years after vaccination with OrthopoxVac and LSV, the VNA levels were notably higher in all vaccinated volunteer groups than they were in the NC group, with no significant differences observed in VNA titers between the compared vaccinated groups.

Analysis of a NC group patient sera via plaque inhibition reaction yielded a GMT VNA value of 1 : 7.

In the experimental groups of phase II/III clinical studies 1.5 years after vaccination, the number of volunteers with VNA titers of ≥ 1 : 10 was 60.0% when OrthopoxVac was administered once at a dose of 10^7^ PoFU and 73.3% when vaccinated twice at a dose of 10^6^ PoFU. The VNA titers of all volunteers vaccinated with LSV was above 1 : 10.

Within the same groups, the proportion of volunteers with VNA titers of ≥ 1 : 10 after 3 years was 53.3% following a single OrtopoxVac vaccine immunization at a dosage of 10^7^ PoFU and 57.1% following a double immunization at a dosage of 106 PoFU, which suggests a gradual decrease in VNA titer over time post-vaccination. VNA titers above 1 : 10 were observed in all LSV-inoculated participants.

The number of volunteers enrolled in phase I clinical studies with VNA titers of ≥ 1 : 10 after 5 years was 77.8% when OrthopoxVac was administered once at a dose of 10^7^ PoFU and 67.7% when vaccinated twice at a dose of 106 PoFU. The number of volunteers vaccinated by the two-stage method with the first-generation vaccine with VNA titers of 1 : 10 or more after 5 years stood at 88.9% (*Fig. 4*).

At 3 years and 5 years after immunization, significant reductions in VNA levels were observed in groups of individuals double-vaccinated with OrthopoxVac at a dose of 106 PoFU compared to the levels determined 1.5 years after vaccination (*Fig. 4B*). In groups vaccinated with a single dose of OrtopoxVac at 10^7^ PoFU, some decrease in VNA titers was observed after 3 and 5 years, with these not significantly different from the titers at 1.5 years (*Fig. 4A*).

No significant differences in VNA titers were found between the groups of patients vaccinated using the two-step method (Inactivated smallpox vaccine OspaVir followed by LSV) at 1.5, 3, and 5 years (*Fig. 4C*).

**Evaluation of T cell anti-smallpox immunity**

The cell-mediated immune response was determined using an intracellular cytokine staining protocol, which detects specific T cells based on their ability to produce cytokines, including IFN-γ, TNF, and IL-2, after costimulation of peripheral blood mononuclear cells (PBMCs) *ex vivo *with the VACΔ6 strain of VACV (see the Experimental Section).

A cytometric analysis of PBMC samples revealed the presence of VACV-specific T helper (CD4^+^) and cytotoxic T lymphocytes (CD8^+^) 1.5 years post-vaccination. After a 20-h stimulation of PBMCs with the VACΔ6 VACV strain, an increase in the number of CD4^+^IFN-γ+ and CD8^+^IFN-γ+ T-cells was observed. Up to 80–90% of the antigen-specific cells were positive for triple (CD4^+^IFN-γ+TNF+IL-2+) or double (CD8^+^IFN-γ+TNF+) cytokine expression.

**Fig. 5:**
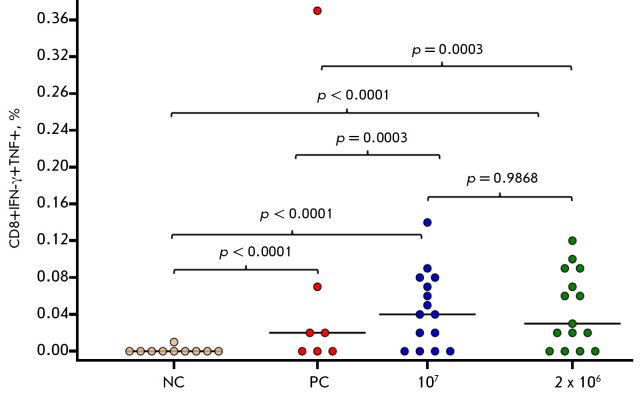
The percentage of VACV-specific CD8^+^ T cells producing FN-γ and TNF in PBMC samples from volunteers vaccinated with the smallpox vaccine in clinical studies 1.5 years after vaccination. NC group (comparison group, negative control) – volunteers not vaccinated with smallpox vaccines, with no contact with patients vaccinated with smallpox vaccines, and who did not work with viruses of the genus Orthopoxvirus; PC group (positive control) – volunteers vaccinated by the two-stage method with the smallpox-inactivated OspaVir vaccine and after 7 days with the live smallpox vaccine based on the L-IVP strain (Microgen); 10^7^ group – volunteers vaccinated once intradermally with the OrthopoxVac vaccine at a dose of 10^7^ PoFU/0.2 mL; 2 × 10^6^ group – volunteers vaccinated twice with a 28-day interval, intradermally at a dose of 10^6^ PoFU/0.2 mL. The significance of the differences between the groups was determined by the F criterion. Each point represents a single volunteer

VACV-specific CD8^+^ T-cells were detected in most volunteers from the groups vaccinated with OrtopoxVac (both single dose 10^7^ PoFU and double dose 10^6^ PoFU). Up to 90% of the CD8^+^IFN-γ+TNF+cell population was negative for the CD57 marker, indicating that these T-cells had not reached a state of terminal differentiation/ exhaustion. In both groups immunized with OrthopoxVac, the level of CD8^+^IFN-γ+TNF+ cells significantly exceeded the corresponding values in the positive control group – volunteers immunized with a first-generation smallpox vaccine (*Fig. 5*).

**Fig. 6:**
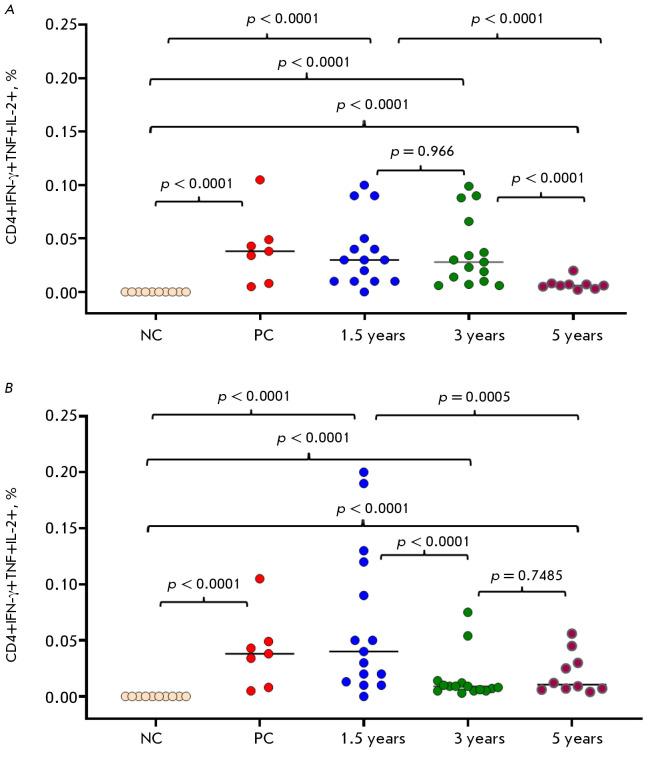
Distribution of smallpox virus-specific polyfunctional CD4^+^ T cells by expression of CCR7 (CD197) and CD45RA markers, 1.5 years after vaccination with smallpox vaccines

A f t e r 1 . 5 ye a r s , t h e q u a nt i t y o f CD4^+^IFN-γ+TNF+IL-2+ T-helper cells within both volunteer groups vaccinated with OrthopoxVac presented no statistically significant differences when compared to the group inoculated with the first-generation live smallpox vaccine (LSV) (*Fig. 6*).

Additionally, the expression of the memory markers CCR7 (CD197) and CD45RA was analyzed in VACV-specific CD4^+^ and CD8^+^ T cells. The effector memory T_EM_ cells (CCR7-CD45RA-) dominated the VACV-specific CD4^+^ T-cell population, with a proportion ranging from 80–90%, followed by central memory T_CM_ cells (CCR7+CD45RA-) at 5–10%, terminally differentiated effector memory T_EMRA_ cells (CCR7-CD45RA+) at 2–5%, and naïve T cells (CCR7+CD45RA+) representing just 1% (*Table 1*).

**Table 1:** Distribution of smallpox virus-specific polyfunctional CD4+ T cells by expression of CCR7 (CD197) and CD45RA markers, 1.5 years after vaccination with smallpox vaccines

Groups	Serum No.	TCM central memory T cells CCR7+CD45RA	Naïve T cells CCR7+CD45RA+	T_EM_, effector memory T cells CCR7-CD45RA+	T_EMRA_ CCR7-CD45RA+
OrthopoxVac 10^7^ (single dose)	0-4-12	6.8 ± 3.6	ND^*^	89.4 ± 4.1	3.8 ± 0.6
	0-4-17	7.5 ± 0.5	1.7 ± 0.8	28.0 ± 1.0	62.8 ± 1.8
	0-4-8	14.7 ± 6.4	ND	85.3 ± 6.4	ND
	0-4-15	8.2 ± 0.3	ND	87.0 ± 0.3	4.7 ± 0.6
	0-4-3	5.3 ± 0.2	0.6 ± 0.8	85.2 ± 3.1	8.9 ± 4.1
	0-4-16	7.2 ± 0.1	ND	92.5 ± 0.7	ND
	0-4-10	5.9 ± 2.6	ND	90.0 ± 3.3	4.2 ± 5.9
	0-4-11	6.0 ± 2.9	ND	92.1 ± 2.9	2.0 ± 0.1
	0-4-13	9.1 ± 0.5	ND	88.7 ± 0.1	2.2 ± 0.3
	0-4-14	4.2 ± 5.9	ND	92.0 ± 0.5	3.8 ± 0.4
	0-4-1	6.1 ± 2.1	0.5 ± 0.7	88.1 ± 0.3	5.3 ± 1.1
	0-4-2	7.8 ± 4.6	ND	89.9 ± 1.4	2.3 ± 3.2
	0-4-5	4.4 ± 1.2	ND	91.2 ± 4.1	4.4 ± 2.9
	0-4-9	13.8 ± 3.2	ND	84.2 ± 3.2	2.0 ± 0.1
	0-4-6	4.9 ± 1.0	ND	93.1 ± 2.0	2.1 ± 2.9
OrthopoxVac 10^6^ (double dose)	0-5-3	5.6 ± 0.9	ND	88.8 ± 1.8	5.6 ± 0.9
	0-5-16	4.9 ± 0.9	ND	85.3 ± 2.2	9.8 ± 1.1
	0-5-4	14.0 ± 2.2	ND	84.9 ± 3.7	1.1 ± 0.1
	0-5-5	8.2 ± 2.5	1.4 ± 2.0	80.8 ± 17.3	8.6 ± 1.6
	0-5-6	3.8 ± 1.4	ND	95.7 ± 4.7	ND
	0-5-8	5.3 ± 0.8	2.9 ± 4.2	81.1 ± 6.5	10.6 ± 1.1
	0-5-9	8.8 ± 2.3	0.3 ± 0.4	87.3 ± 2.1	3.6 ± 2.1
	0-5-11	6.1 ± 3.4	0.6 ± 0.3	90.1 ± 2.0	3.2 ± 0.3
	0-5-12	7.6 ± 6.4	ND	90.9 ± 8.6	1.5 ± 1.1
	0-5-17	12.7 ± 0.8	1.3 ± 1.9	84.8 ± 2.3	1.1 ± 0.1
	0-5-18	10.4 ± 2.0	3.3 ± 0.1	75.8 ± 0.8	10.5 ± 1.1
	0-5-1	6.8 ± 0.8	ND	86.2 ± 3.5	7.0 ± 2.7
	0-5-2	7.3 ± 0.4	1.0 ± 0.1	91.2 ± 0.4	0.5 ± 0.7
	0-5-10	6.3 ± 0.1	0.8 ± 0.4	92.4 ± 0.3	0.5 ± 1.1
	0-5-7	9.5 ± 2.1	0.4 ± 2.1	86.0 ± 1.5	2.0 ± 2.8
Positive control	0-2-34	6.3 ± 1.3	5.3 ± 0.9	77.1 ± 3.2	11.4 ± 1.2
	0-2-32	1.9 ± 0.8	ND	93.1 ± 1.1	5.1 ± 2.3
	0-2-2	12.2 ± 0.2	1.2 ± 0.1	72.5 ± 2.8	14.1 ± 2.1
	0-2-3	9.0 ± 2.7	2.2 ± 0.1	59.6 ± 1.6	29.3 ± 3.3
	0-2-30	7.3 ± 2.8	ND	90.9 ± 5.4	1.9 ± 0.6
	0-2-36	4.2 ± 1.2	1.7 ± 0.2	79.6 ± 4.4	14.6 ± 3.1

^*^Note: ND – not detected (below the sensitivity level of the method).

The percentage of T_EM_ (CCR7-CD45RA-) in the VACV-specific CD8^+^ T cell population was about 20%, and the percentage of T_EMRA_ (CCR7-CD45RA+) was up to 80%.

At the three- and five-year point following immunization with LSV and OrthopoxVac, the level of specific CD8^+^ T cells in the PBMC preparations from volunteers, after VACV costimulation, was undetectable using this method.

In volunteers immunized with the OrtopoxVac at 10^7^ PoFU, the production of CD4^+^IFN-γ+TNF+IL-2+ T-helpers remained at its initial level at the 3-year point but decreased significantly by the 5-year point (*Fig. 6A*). For volunteers administered two doses of the OrthopoxVac vaccine (10^6^ PoFU), a significant reduction in VACV-specific T-helper cell levels was observed by the third year following vaccination, which persisted up to five years post-vaccination (*Fig. 6B*).

**Table 2:** Distribution of poxvirus-specific polyfunctional CD4+ T cells by expression of CCR7 (CD197) and CD45RA markers, 3 years after vaccination with smallpox vaccines

Groups	Serum No.	TCM central memory T cells CCR7+CD45RA	Naïve T cells CCR7+CD45RA+	T_EM_, effector memory T cells CCR7-CD45RA+	T_EMRA_ CCR7-CD45RA+
OrthopoxVac 10^7^ (single dose)	155	6.6 ± 2.2	0.5 ± 0.1	87.2 ± 0.9	5.8 ± 0.7
	158	5.8 ± 3.0	ND^*^	91.6 ± 6.7	2.6 ± 0.6
	164	12.9 ± 4.1	ND	87.1 ± 4.1	ND
	166	7.5 ± 1.0	ND	82.8 ± 4.7	6.7 ± 1.7
	199	7.0 ± 0.2	0.6 ± 0.2	86.9 ± 0.1	5.6 ± 0.1
	206	11.3 ± 5.2	ND	86.8 ± 6.1	1.9 ± 0.9
	209	10.2 ± 0.5	ND	86.4 ± 1.5	1.3 ± 1.8
	216	10.9 ± 2.7	0.7 ± 0.1	85.4 ± 2.4	3.1 ± 0.4
	222	10.8 ± 2.2	ND	86.1 ± 0.8	3.1 ± 3.0
	223	14.2 ± 1.9	1.7 ± 0.6	82.3 ± 4.0	1.8 ± 2.5
	229	7.7 ± 0.1	ND	54.6 ± 0.5	36.7 ± 0.4
	246	8.8 ± 2.9	ND	87.9 ± 7.5	3.3 ± 1.6
	249	7.6 ± 1.5	1.1 ± 0.3	85.7 ± 0.9	5.6 ± 2.1
	246	11.0 ± 1.0	0.8 ± 1.1	85.6 ± 5.4	2.7 ± 0.5
	259	8.0 ± 0.6	ND	89.7 ± 1.4	2.3 ± 0.7
OrthopoxVac 10^6^ (double dose)	059	9.2 ± 3.1	2.2 ± 1.1	82.6 ± 3.8	6.0 ± 0.2
	089	3.5 ± 4.9	ND	88.8 ± 3.6	7.8 ± 2.5
	095	6.1 ± 1.2	2.6 ± 0.7	89.6 ± 0.1	1.7 ± 0.3
	098	7.1 ± 0.1	ND	92.4 ± 9.4	0.1 ± 0.1
	108	3.8 ± 1.7	0.6 ± 0.3	85.8 ± 2.1	9.8 ± 2.1
	106	8.0 ± 0.2	ND	88.2 ± 2.5	3.8 ± 1.2
	105	6.0 ± 1.3	1.1 ± 0.4	90.6 ± 1.2	2.3 ± 0.1
	104	7.1 ± 1.2	0.6 ± 0.2	88.4 ± 1.2	3.9 ± 0.2
	103	9.0 ± 2.8	ND	81.2 ± 1.7	9.8 ± 2.9
	178	4.1 ± 3.2	0.9 ± 0.3	87.6 ± 4.9	7.4 ± 2.4
	177	9.6 ± 1.3	ND	90.8 ± 9.3	2.6 ± 0.1
	109	6.7 ± 0.1	ND	86.4 ± 1.8	6.8 ± 1.8
	255	6.5 ± 0.7	ND	90.5 ± 0.7	3.0 ± 0.1
	256	7.3 ± 0.4	ND	91.8 ± 0.4	2.0 ± 0.1
	257	6.0 ± 1.4	ND	93.9 ± 7.1	2.5 ± 1.3
Positive control	BLV	7.1 ± 1.6	0.5 ± 0.7	90.5 ± 3.9	1.9 ± 0.5
	DGV	5.9 ± 0.8	0.7 ± 0.1	75.7 ± 0.6	17.7 ± 1.3
	RAS	5.0 ± 0.1	0.4 ± 0.5	79.3 ± 2.9	15.3 ± 2.4
	GTA	13.0 ± 2.6	0.3 ± 0.4	83.6 ± 3.4	3.2 ± 0.8
	FEN	7.7 ± 2.9	ND	68.3 ± 9.5	24.0 ± 1.4
	NIN	9.8 ± 1.6	2.9 ± 1.2	87.2 ± 1.4	ND
	LMP	7.9 ± 1.7	3.7 ± 0.1	67.1 ± 1.6	21.3 ± 3.4

^*^Note: ND – not detected (below the sensitivity level of the method).

Three years post-immunization, the VACVspecific CD4^+^ T-cell population in all volunteer groups predominantly comprised effector memory T_EM_ cells (CCR7-CD45RA-) at 80–90%, central memory TCM cells (CCR7+CD45RA-) at 5–10%, T_EMRA_ (CCR7-CD45RA+) at 2–10%, and up to 1% of naïve T cells (CCR7+CD45RA+) (*Table 2*). Cell distribution based on the memory markers CCR7 (CD197) and CD45RA was typical of both volunteers vaccinated with the fourth-generation OrthopoxVac vaccine and those vaccinated using the two-stage method involving inactivated smallpox vaccine and first-generation LSV.

## DISCUSSION

The primary challenge when creating a novel smallpox vaccine is the necessity to attenuate the virulence of the VACV vaccine strain while ensuring an adequate and durable humoral and cellular immune response. The standards for determining the degree of immunity generated in humans following smallpox vaccination and that confers complete protection against orthopoxvirus infections have yet to be set. Only a limited number of publications have sought to define such criteria.

Historically, the first criterion for assessing the immune response to the Variola virus infection or VACV vaccination has been to determine VNA levels in the patients’ sera. In a study by Mack et al. [17], individuals with a VACV VNA titer below 1 : 32 were found to be more prone to infection upon coming into contact with each other (20% of contacts became ill), in contrast to those with a VNA titer of 1 : 32 or higher (1% of contacts became ill). It was also observed that during an epidemic, smallpox was contracted by 14% of exposed, unvaccinated patients with VNA to VACV titers of < 1 : 20, whereas patients with VNA titers ≥1 : 20 did not contract the disease [18]. However, it is important to note that no previously vaccinated patients, including those with VNA titers < 1 : 10, not contracted smallpox by interacting with other patients. The protective power of a single injectable vaccinia immune globulin preparation has led to the conclusion that even low levels of VNA can provide a sufficient degree of protection against smallpox [19].

In addition to VNA, the cellular immune response plays a vital role in the defense against smallpox [15, 19, 21, 21]. However, at the time of smallpox eradication, the methodologies for assessing cell-mediated immune responses had not yet advanced that much. Therefore, criteria for a protective level of the T-cell response to smallpox vaccination have yet to be established [16, 22].

T cells are critical in the early stages of identification and suppression of viral infections, as well as in supporting B cells to produce antibodies. Given the crucial role of T cells, they represent a vital target in evaluating immune responses to an infection or vaccination.

Immunization with the smallpox vaccine generates enduring cell-mediated immune responses via CD4^+^ and CD8^+^ T cells, with peak numbers observed between two and four weeks following vaccination, followed by a drop, and ultimately a sustained, stable crop of memory T cells [23, 24]. It should be noted that the population of memory CD8^+^ T cells declines faster than that of memory CD4^+^ T cells [25]. The need for CD4^+^ T cells for purposes of protection is demonstrated by the absence of VACV-specific antibodies in animals that lack CD4^+^ T cells [26, 27]. Additionally, CD4^+^ T cells are vital for optimal cytotoxic T-lymphocyte functioning and immunologic memory formation [28].

A significant challenge in demonstrating the effectiveness of novel smallpox vaccines lies in the inability to directly prove that newly developed vaccines elicit protective immunity against smallpox in humans. Given the elimination of smallpox, assessing the efficacy of new vaccines against a naturally occurring disease is impossible. Alternatively, new vaccines undergoing clinical studies should be assessed against existing benchmarks and compared to the first-generation smallpox vaccines utilized in the initial eradication drive [16, 23].

On November 11, 2022, the Ministry of Health of the Russian Federation authorized OrthopoxVac, the first fourth-generation attenuated smallpox vaccine (a live culture vaccine for the prevention of smallpox and related orthopoxvirus infections based on the vaccinia virus). This vaccine was designed using the L-IVP VACV strain used in Russia as a first-generation smallpox vaccine (live smallpox vaccine) [2, 11]. A number of the genes in this strain were targeted for inactivation using genetic engineering methods. These included the genes encoding the gamma-interferonbinding protein (*B8R*), the complement-binding protein (*C3L*), the Bcl-2-like inhibitor of apoptosis (*N1L*), hemagglutinin (*A56R*), thymidine kinase (*J2R*) and the* A35R *gene, whose protein product inhibits the presentation of antigens by major histocompatibility complex class II, the immune priming of T-lymphocytes, and the subsequent synthesis of chemokines and cytokines. The VACV strain thus created was given the name VACΔ6 [11]. Following a series of preclinical studies [29] and subsequent phases I and II/III clinical studies (CS), the OrthopoxVac vaccine was deemed to be a safe and weakly reactogenic preparation, with immunologic activity comparable to that of the original Russian first generation smallpox vaccine.

At 60, 90, and 180 days after a double 106 PoFU dose of OrthopoxVac vaccine immunization, volunteer sera exhibited GMT VNA values of 79.4, 75.9, and 69.2, respectively. Following a single 107 PoFU injection of OrthopoxVac, the respective values were 138.0, 31.7, and 31.6. The GMT VNA values in sera from volunteers who had received the two-step vaccination regimen with the first-generation vaccine were 104.7, 52.5, and 63.1 at the specified time periods.

As is clear, a gradual decrease in VNA titers was observed over a 6-month period in all the vaccinated volunteer groups under study.

It should be noted that OrthopoxVac possesses higher immunogenicity compared to the third-generation MVA smallpox vaccine, which has become widespread in recent years [30]. An optimal immune response necessitates a two-dose administration of this non-replicating, attenuated vaccine. A clinical study has indicated that in the sera of two groups of volunteers immunized twice with liquid or lyophilized MVA preparations, the GMT values of VNA first stood at 45.2 and 77.6, respectively, 14 days after the second administration, before falling to 10.2 and 11.7, respectively, by day 180 [31].

According to the current guidelines in Russian “Conducting smallpox vaccination. MU 3.3.1.2044-06,” the next revaccination of people from risk groups with the first-generation vaccine, except for those directly working with smallpox and monkeypox viruses, happens after 5 years. Those working with smallpox and monkeypox viruses are revaccinated after 3 years.

Due to the altered genetic program of the VACV strain VACΔ6, as opposed to the original L-IVP strain, it was imperative to examine the length and strength of the post-vaccination immune response in the individuals who had received the OrthopoxVac vaccine. Previous investigations had only measured the development of the humoral immune response within six months post-vaccination, without assessing the production of VACV-specific CD4^+^ and CD8^+^ T-lymphocytes. This study has evaluated the humoral and T-cell responses to intradermal OrthopoxVac injections in phase II/III CS participants at the 1.5- and 3-year time points and in phase I CS participants at the 5-year time point, as well as compared them to those who had received the first-generation smallpox vaccine.

The humoral immune response was assessed using standard methods: ELISA for determining the specific antibody titer and VACV neutralization reaction on the cell culture.

ELISA-based assessment of VACV-specific antibody titers (*Fig. 3*) demonstrated notable inter-individual variability within each cohort, aligning with previously reported findings and potentially attributable to immune system-related genetic polymorphisms [13, 32, 33]. It is of significance that a notable VACV-specific humoral response was recorded both three and five years after immunization with the first-generation vaccine and the created fourthgeneration OrthopoxVac vaccine. At the same time, no significant differences were observed between the compared groups.

Following a period of 1.5 years post-immunization with the OrthopoxVac vaccine, VNA titers exhibited a drop in certain patients (*Fig. 4A,B*). It is worth noting that, when OrthopoxVac was administered at a dose of 107 PoFU, no significant differences were observed among the groups at 1.5, 3, and 5 years (*Fig. 4A*). However, VNA titers had significantly decreased after 1.5 years in the groups that received two immunizations at a dose of 106 PoFU (*Fig. 4B*).

Variations in the proportions of volunteers exhibiting VNA titers exceeding 1 : 10 at the 3- and 5-year post-vaccination intervals can be attributed to the differing volunteer cohorts involved in the phase II/III and phase I clinical studies, respectively.

Cytometric analyses performed on PBMC preparations of vaccinated volunteers 1.5 years after immunization revealed VACV-specific T cells, including both T helper (CD4^+^) and cytotoxic T lymphocytes (CD8^+^). The T-helper cells elicited a more significant cell-mediated immune response than the cytotoxic T-lymphocytes. The loads of CD8^+^ cells in both groups of volunteers inoculated with OrthopoxVac were notably higher than those in the positive control group (*Fig. 5*), which presumably can be attributed to the differences in the genetic programs of the recombinant VACΔ6 and initial L-IVP VACV strain.

Regardless of the dosage or administration method, the OrthopoxVac vaccine generated an effective T-helper cell-mediated immune response to orthopoxviruses 1.5 years after vaccination (*Fig. 6*).

Three years post-vaccination, the study of intracellular cytokines in volunteer PBMC preparations, costimulated with the attenuated VACΔ6 VACV strain, revealed virus-specific T-cell immune responses exclusively in T-helper cells, regardless of whether first- or fourth-generation vaccines were used. VACVspecific cytotoxic T lymphocytes (CD8^+^) were detected in only one volunteer after twice administration of OrthopoxVac at a dose of 10^6^ PoFU.

T-helper cells specific to VACV were found 3 and 5 years following OrthopoxVac vaccination. However, the strength of the immune response differed depending on the dosage and method of administration. In volunteers immunized with OrthopoxVac at a dose of 10^7^ PoFU, the T-helper response stayed relatively elevated for three years before it substantial dropped. If patients had received two doses of the vaccine at 10^6^ PoFU, a substantial reduction in T-helper cells was observed after 1.5 years (*Fig. 6*). The bulk of the specific T cells displayed the characteristics of memory effector cells (*Table 1* and *Table 2*), suggesting they were in active interaction with the antigen.

Following the administration of the fourth-generation smallpox vaccine OrthopoxVac, all our volunteers, regardless of dosage or method of administration, experienced a cell-mediated immune response to VACV at the three and five-year intervals.

The findings here indicate that a single intradermal injection of the OrthopoxVac vaccine, at a dosage of 10^7^ PoFU, triggers a significant and specific immune response, including both humoral and T-cell immunity, that persists for at least three years. Additional clinical studies are warranted to establish the most effective revaccination strategy with the OrthopoxVac vaccine, with the goal of achieving prolonged immunity against orthopoxvirus infections.

## Abbreviations

VNA: virus neutralizing antibodies
VACV: vaccinia virus
LSV: live smallpox vaccine
WHO: World Health Organization
CS: clinical studies
PFU: plaque forming unit
PoFU: pock forming unit
GMT: geometric mean titer
PBMC: peripheral blood mononuclear cells
